# Antioxidant Properties of “Natchez” and “Triple Crown” Blackberries Using Korean Traditional Winemaking Techniques

**DOI:** 10.1155/2017/5468149

**Published:** 2017-06-21

**Authors:** Youri Joh, Niels Maness, William McGlynn

**Affiliations:** ^1^Robert M. Kerr Food & Agricultural Products Center, Oklahoma State University, Stillwater, OK 74078, USA; ^2^Department of Horticulture and Landscape Architecture, Oklahoma State University, Stillwater, OK 74078, USA

## Abstract

This research evaluated blackberries grown in Oklahoma and wines produced using a modified traditional Korean technique employing relatively oxygen-permeable earthenware fermentation vessels. The fermentation variables were temperature (21.6°C versus 26.6°C) and yeast inoculation versus wild fermentation. Wild fermented wines had higher total phenolic concentration than yeast fermented wines. Overall, wines had a relatively high concentration of anthocyanin (85–320 mg L^−1^ malvidin-3-monoglucoside) and antioxidant capacity (9776–37845 *µ*mol Trolox equivalent g^−1^). “Natchez” berries had a higher anthocyanin concentration than “Triple Crown” berries. Higher fermentation temperature at the start of the winemaking process followed by the use of lower fermentation/storage temperature for aging wine samples maximized phenolic compound extraction/retention. The Korean winemaking technique used in this study produced blackberry wines that were excellent sources of polyphenolic compounds as well as being high in antioxidant capacity as measured by the Oxygen Radical Absorbance Capacity (ORAC) test.

## 1. Introduction

Winemaking and wine consumption are becoming more popular as they are known to provide health beneficial products that are high in antioxidants [1–3]. Blackberry (*Rubus *spp.) wines are good sources of antioxidants because they contain relatively high concentrations of anthocyanins and other phenolic compounds [4–6]. Fermentation processes have been shown to increase the level of antioxidant activity by facilitating the extraction of anthocyanins and other phenolic compounds from the pomace and by forming new polymerized pigments and polyphenols [7]. The Korean traditional wine processing method, which typically employs wild microorganisms for fermentation, may provide different types and levels of health related compounds compared to common grape wine production methods.

Blackberry phenolic composition has been shown to vary on the basis of growing temperature, growing season, geographic location, maturity at harvest, environmental stress, soil type, UV light exposure, hydrophobicity of compounds, genetics, extraction/processing methods, and processing storage conditions [3, 8–15]. Relatively little research has been done on “Natchez” and “Triple Crown” blackberries, cultivars that are suitable for growing in the Midwest section of the United States. The suitability of these blackberries for winemaking of phenolic compounds of wines made from these berries has not previously been studied. The part of the research was presented in ASEV National Conference before, but full information was provided in this research paper [16].

The objectives of this study were to evaluate the winemaking potential of “Natchez” and “Triple Crown” blackberries grown in Oklahoma as well as to examine the chemical properties of phenolic compounds of blackberry wines made using variations on traditional Korean winemaking techniques. The fermentation parameters were fermentation temperature, that is 21.6°C and 26.6°C, and yeast inoculation fermentation versus wild fermentation. The pH, % soluble solids, titratable acidity, and % alcohol of berries and wines were assayed to assess basic quality parameters. Also, the chemical properties of the berries and wines were evaluated by quantifying their total phenolic concentration, anthocyanin concentration, and antioxidant capacity.

## 2. Materials and Methods

### 2.1. Blackberry Collection and Storage

Fruit from two blackberry cultivars (*Rubus *spp.), “Natchez” and “Triple Crown,” were collected from the Oklahoma Agricultural Experiment Stations, Cimarron Valley Research Station (Perkins, OK, USA). Blackberries were collected after they turned fully purplish black over a period of two years, 2011 and 2012. All blackberries were hand-harvested starting from the third week of May and ending about the third week of July. The ripening time of “Natchez” berries was approximately one month earlier than that of “Triple Crown” berries. During the harvest period, the berries were collected every other day. Blackberries were placed into polyethylene bags and placed into the freezer (−15°C) within one hour of harvest for storage and subsequent experimental use.

### 2.2. Preparation of Juice Sample

Frozen whole blackberries were placed in a refrigerator at about 4°C for a day and then held at room temperature until they came to temperature equilibrium in about 3 to 4 hours. Fresh juice samples were collected by manually pressing 100 to 150 blackberries against a 2 mm mesh screen. Juice samples of at least 100 mL were collected into 120 mL brown amber bottles for future analysis.

### 2.3. Korean Traditional Blackberry Winemaking Process

#### 2.3.1. Prefermentation Handling

**Figure 1:**
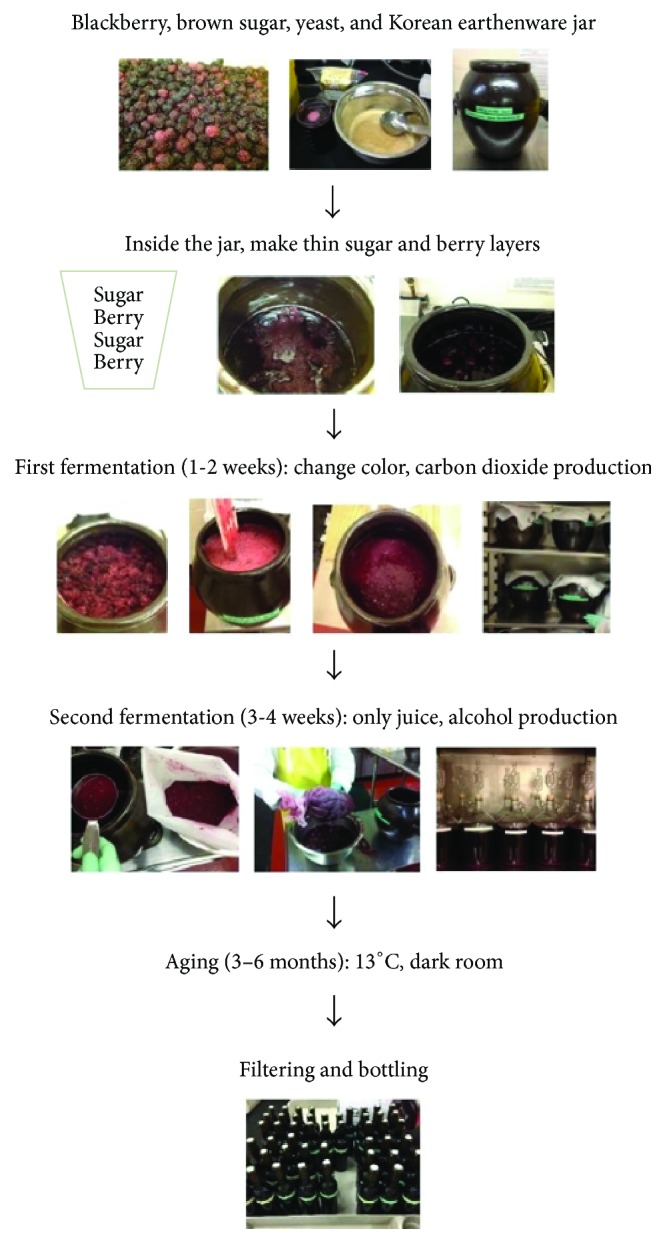
Overview of Korean traditional winemaking process.

A modified combination of Korean traditional winemaking techniques was used in this research [17–19].  Figure 1 shows an overview of the Korean traditional winemaking process [20]. Prewashed 12 L traditional Korean earthenware jars (Sin-il Earthenware, Inchon, South Korea) were used as fermentation vessels. Blackberries (≈4.5 kg) thawed as previously described were placed in each Korean earthenware jar and ≈20% raw brown sugar (Cumberland Packing Co., Brooklyn, NY, USA) by blackberry weight was added. Alternating layers (≈5 cm thick) of blackberries and sugar were laid down in each jar such that the jars were ≈2/3 full by volume.

After filling the blackberries and sugar into the Korean earthenware jars, the jars were covered with thin paper (breathable) secured around the neck of the jar with a string. The treatment factors applied were two cultivars (“Natchez” and “Triple Crown”), two fermentation temperatures (26.6°C and 21.6°C), and two fermentation microflora (no added yeast and added yeast). For the yeast, 5 g of Enoferm L2226 (Scott Laboratories Inc., Petaluma, CA, USA) in 50 mL water was added in each jar.

Three Korean earthenware jars were used for each treatment combination; each jar was considered an experimental unit for purposes of statistical analyses. Thus, each treatment combination was replicated three times. An environmental chamber (Ultimate Hot Pack Inc., Lander, WY, USA) was used for temperature control.

#### 2.3.2. Fermentation

A two-part fermentation process was used for all samples [20]. The first fermentation took 1 to 2 weeks. During the first fermentation period, samples were mixed with a spatula every morning and evening to help insure sufficient aeration. The Korean earthenware jar facilitated this process as the container was breathable with many small pores (1 to 20 *µ*m) that allowed relatively more gas transfer into the samples than plastic or glass jars would have done [21]. While active fermentation was under way, the blackberries changed color from purple to pink and the individual berries lost their structural integrity. The end of the first fermentation stage occurred when CO_2_ release rate slowed and the soluble solids concentration dropped below 10° brix.

The second fermentation stage took 3 to 4 weeks. Blackberry skins and seeds were removed using a nylon straining bag (small fine size, 10′′ × 23′′, LD Carlson, Kent, OH, USA). The strained pomace was collected into polyethylene bags and stored in a −15°C freezer for further analysis. The strained juice was then transferred into a second type of fermentation vessel. During the second stage of fermentation, the goal was to limit oxygen contact. For this reason, glass or plastic fermentation vessels were used and each vessel was filled to within ≈4/5 full [19]. The finishing point of the second fermentation stage occurred when no further production of CO_2_ gas was noted via airlock apparatus. Fermentation temperature was controlled at 21.6°C or 26.6°C during both fermentation stages. Triplicate samples were collected into 120 mL brown bottles at the end of 1st and 2nd fermentation and stored in a −15°C freezer for further analysis.

#### 2.3.3. Aging Wine

After the second fermentation stage was complete, the wine was racked (decanted off the sediment at the bottom of the vessel) and filled into 950 mL brown amber glass bottles to the top and the bottles were tightly sealed with screw caps. The wine was stored at 13°C [19] and 100 mL samples were collected once a month into 120 mL brown bottles for three months for further analyses.

### 2.4. Quality Analysis for Whole Blackberry, Juice, and Wine

#### 2.4.1. pH

The pH of the blackberry juice was measured using an Accumet AB 15 pH meter (Buffalo, NY, USA). Duplicate samples were measured and averaged for each replication.

#### 2.4.2. Soluble Solids

Blackberry juice sugar concentration was estimated as percent soluble solids using a Leica Auto ABBE refractometer (Buffalo, NY, USA). Duplicate samples were measured and averaged for each replication.

#### 2.4.3. Titratable Acidity

The titratable acidity of blackberry juice and wine samples was measured manually using 0.1 N sodium hydroxide (Arcos Organics, Fair Lawn, NJ, USA) as per the method described in Joh [20]. Two duplicate readings were taken from each blackberry juice and wine sample and then averaged.

#### 2.4.4. Percent Alcohol

The percent alcohol (w/w) of wine samples was measured using an Alcolyer Wine M (Anton Paar, Ashland, VA, USA). This instrument uses a patented method (US 6,690,015; AT 406711) based on near infrared (NIR) spectroscopy to determine the alcohol content in a highly alcohol-specific wavelength range between 1150 nm and 1200 nm [22]. Samples of the aged wines were collected into 60 mL brown glass bottles. A volume of ≈30 mL of wine per sample was used in the analysis. Two duplicate readings were taken from each wine sample and averaged.

### 2.5. Antioxidant Activity Analyses

#### 2.5.1. Modified Harbertson-Adams Assay

*(1) Total Phenolic Concentrations*. A volume of 75 *µ*L of blackberry juice or wine and 800 *µ*L resuspension buffer was add to a reduced volume cuvette and then held for 10 minutes at room temperature. Samples were read at 510 nm to generate a value for the iron-reactive phenolics background. In the same cuvette, 125 *µ*L of ferric chloride solution was added and held for another for 10 minutes at room temperature. Samples were read at 510 nm to generate a final value for the iron-reactive phenolics concentration [20].

*(2) Total Anthocyanin Concentrations*. A volume of 400 *µ*L of model wine, 100 *µ*L of blackberry juice or wine sample, and 1 mL of anthocyanin buffer was added to a reduced volume cuvette and then held for 5 minutes at room temperature. Samples were then read at 520 nm [20].

All samples were measured in duplicate. Final value calculations were made using the Skogerson-Boulton Model Assay Input spreadsheet (Boulton Research: Skogerson-Boulton Model Assay Input v.1.3) [20].

#### 2.5.2. Oxygen Radical Absorbance Capacity (ORAC) Assay

All blackberry juice, wine, and pomace samples were added at a ratio of 1 : 2000 (v/v) to phosphate buffer prior to being tested for antioxidant capacity using a slightly modified version of the Oxygen Radical Absorbance Capacity (ORAC) assay described in Huang and others [23]. The details of the method used may be found in Joh [20]. The final results of the ORAC assay were calculated as *µ*mol Trolox equivalent (TE) per gram of blackberry juice, wine, or pomace. All samples were measured in duplicate.

### 2.6. Statistical Analysis

Statistical Analyses were performed using SAS 9.3 (SAS institution, Cary, NC). For all analyses, an analysis of variance (ANOVA) for each set of data was conducted using a three-factor factorial treatment scheme in a completely randomized design with repeated measures. Means were separated using least significant differences (LSD) with a 95% confidence interval (*p* < 0.05).

## 3. Results and Discussions

### 3.1. Quality Analysis for Whole Blackberry Juice and Wine

#### 3.1.1. pH

The mean pH values of blackberry juice samples made from berries harvested in 2011 and 2012 are shown in Table 1. Values ranged from 2.88 to 3.15. The pH values of blackberry wine samples are shown in Table 2. Values ranged from 2.60 to 3.12.

The pH values in blackberry juice and wine matched several previous studies [7, 24–26]. Some researchers found higher pH values, from 3.2 to 4.2, likely due to differences observed among cultivars, growing locations, and/or berry ripeness [27–29].

“Triple Crown” berries showed significant differences between years. It appears that weather condition such as amount of rainfall affected the pH of the berries. The acidity level in blackberries has been observed to decrease under warmer, drier weather conditions [27]. In 2012, the average rainfall of July was 0.2 cm compared to the average rainfall of 1.9 cm in 2011 [30]. The drier conditions in 2012 may have helped to ripen “Triple Crown” berries faster that year and provided less acidic berries.

Wine samples showed statistically significant differences between fermentation temperatures (*p* < 0.05). Within the cultivar and inoculation treatment, lower fermentation temperature samples had higher pH values than higher fermentation temperature samples. However, comparing cultivars within inoculation treatments, only samples at the higher fermentation temperature had statistically significant differences: “Triple Crown” berries had higher pH values than “Natchez” berries. Also, “Triple Crown” berries showed that yeast-inoculated samples had higher pH values than wild treatment samples.

#### 3.1.2. Titratable Acidity

Titratable acidity of blackberry juice was expressed as % malic acid (MA) and the mean values are shown in Table 1. Observed values ranged from 0.386 to 0.422% MA. The pattern of the results was the same as that seen for pH values. “Triple Crown” berries showed significant difference between years (*p* < 0.05): year 2012 had higher titratable acidity than year 2011.

The mean titratable acidity values of blackberry wines are shown in Table 2. Observed values ranged from 0.350 to 0.420% MA. Our titratable acidity range was similar to the range measured in previous research, from 0.33 to 0.41% MA [8]. Most researchers have recorded somewhat higher titratable acidity values than those observed in the current study [24, 26, 27, 31]. Titratable acidity can be influenced by the weather conditions: lack of sunshine, low temperature, or high rainfall. The relatively low titratable acidity values measured in this study suggest that the berries were less tart than some. However, fermentation likely played a role in the differences observed as well. All samples showed statistically significant differences between fermentation temperatures as shown in Table 2 (*p* < 0.05). Within the cultivars, samples with lower fermentation temperature had higher titratable acidity than samples with higher fermentation temperature. Comparing the two cultivars, “Triple Crown” berries had higher titratable acidity than “Natchez” berries in the higher fermentation temperature samples. In addition, “Triple Crown” berries with yeast inoculation samples showed higher titratable acidity values than wild fermentation.

#### 3.1.3. Soluble Solids

The mean sugar concentrations of blackberry juice samples, expressed as % soluble solids, are shown in Table 1. Values ranged from 10.04 to 12.06%. The soluble solids concentration of blackberry juice observed in this study (Table 1) was about 10 to 12%, which is close to the range seen in other research [32]. However, many previous researchers have reported slightly lower soluble solid concentrations of below 10% [8, 27]. It is well known that environmental condition such as weather and planting location can affect the sugar level of blackberries. Sugar levels in fruits have been shown to be affected by weather conditions leading up to and during harvest [27]. More sunshine and less rain or clouds during berry development could help to increase blackberry sugar concentration as well as the formation of good sugar/acid balance, and this may account for some of the differences in sugar content seen among treatments [1].

Statistically significant differences in soluble solids content were shown between the two years and cultivars (*p* < 0.05). Between the two years, “Natchez” berries in 2011 had higher percent soluble solids values than samples from 2012. “Natchez” berries were harvested in June. According to the Oklahoma Mesonet weather data [30], higher average rainfall was observed in June 2012 (5.5 cm) than in June 2011 (4.3 cm) and this may have led to berries that were less sweet in 2012. Between the cultivars, “Triple Crown” berries in 2012 had higher percent soluble solids than “Natchez” berries in 2012. This may be explained by the fact that “Triple Crown” berries were harvested in July, which had less rainfall (0.2 cm) than June (5.5 cm) in 2012.

#### 3.1.4. Percent Alcohol

The average percent alcohol of blackberry wines is shown in Table 2. The alcohol concentrations measured in the blackberry wines ranged from 13.26 to 15.76%. All treatments except for the “Triple Crown” yeast had 13-14% alcohol concentration, while the “Triple Crown” yeast exceeded 15% alcohol concentration. In general, alcohol percentages were higher than those recorded by some other researchers [28]. Because sugar was added while processing the wine, it is not surprising that a relatively high alcohol concentration was seen in the wines. Other research showed that the alcohol concentration of commercial blackberry wines is about 9 to 15% and the wines were measured at 7 to 24% alcohol [29].

The wines made in this study were well within this range. Alcohol content in wines is a function of the sugar concentration in the starting material, up to the alcohol tolerance of the yeast doing the fermentation, presuming that the fermentation goes to completion. However, other factors may affect fermentation efficiency, such as available fermentable nitrogen and other yeast nutrients.

Within cultivar, inoculation treatment showed statistically significant differences (*p* < 0.05). Yeast-inoculated samples had higher percent alcohol than wild fermentation samples. In this study, “Triple Crown” berries produced more alcohol with yeast inoculation than “Natchez” wines. Since the same ratio of sugar was added based on the total volume of berries used, alcohol should have been produced at a similar rate. In addition, almost all samples showed statistically significant differences between the two cultivars and “Triple Crown” showed higher percent alcohol than “Natchez” berries (*p* < 0.05). The “Triple Crown” berries had somewhat higher starting soluble solids concentration than “Natchez” berries but not high enough to account for the observed final difference in wine alcohol concentration. This suggests that other fermentation factors affected the final alcohol concentrations seen. The “Natchez” berries did not provide favorable condition for yeast growth as “Triple Crown” berries and this may have limited yeast activity in “Natchez” wines. Although the wild fermentation for “Triple Crown” berries had lower alcohol concentration than the yeast inoculation, it was more consistent in terms of final alcohol concentration than “Natchez,” indicating that fermentation conditions may have been better for both yeast and wild microflora in the “Triple Crown” musts.

### 3.2. Antioxidant Activity Analyses

#### 3.2.1. Modified Harbertson-Adams Assay

**Figure 2:**
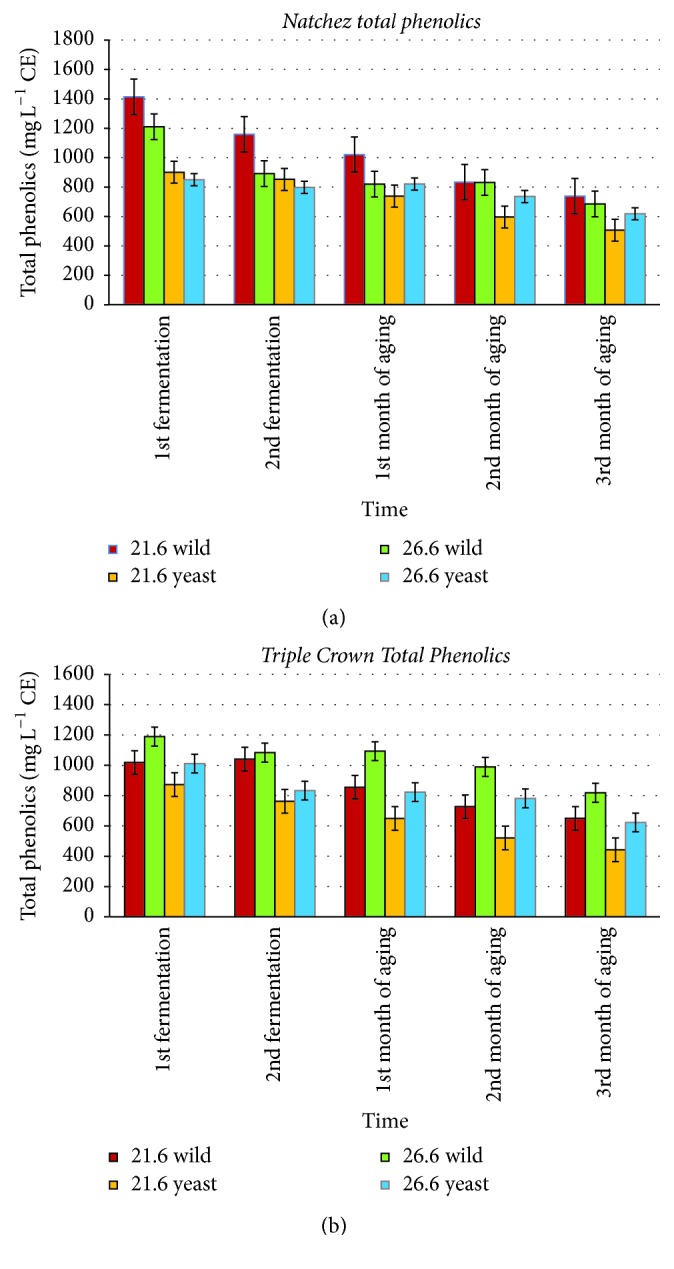
Total phenolic concentrations (*N* = 120) of “Natchez” blackberry (a) and “Triple Crown” blackberry (b) fermented juices and wines. Error bars represent ± standard deviation of the mean (*n* = 2).

*(1) Total Phenolic Concentration*. The total phenolic concentration in blackberry fermented juice and wine, expressed as mg L^−1^ catechin equivalents (CE), is shown in Figure 2. Total phenolic concentrations of blackberry juices and wines were ranged from 440 to 1420 mg L^−1^ CE.

Our results for total phenolic concentrations (Figure 2) were similar to other research [24], showing the concentrations between 601 and 1624 mg L^−1^ CE. Blackberry wine showed a similar lower end for total phenolics with values ranging from 380 to 520 mg L^−1^ CE [33]. This article examined the effect of storage on total phenolics and, similar to our results (Figure 2), the levels of total phenolics decreased over time. Some blackberry wine research [3, 34] had total phenolic concentrations between 1608 and 2836 mg CE L^−1^ which were higher than our results.

Statistically significant differences were seen between cultivars, fermentation temperatures, and inoculation treatments (*p* < 0.05). Fermentation temperature in combination with cultivar type could have affected total phenolic concentrations this study, particularly early in the winemaking process. Higher fermentation temperatures were generally correlated with higher total phenolics concentrations for “Triple Crown” but not “Natchez” wines; this may reflect different extraction kinetics for phenolics in “Triple Crown” berries.

Wild fermented wines were also generally higher in total phenolic concentration than yeast fermented wines. One possible reason for this result is that wild fermentation microorganisms created a fermentation environment that was more conducive to the preservation of phenolic compounds, perhaps by inhibiting phenolic polymerization and complex formation.

**Figure 3:**
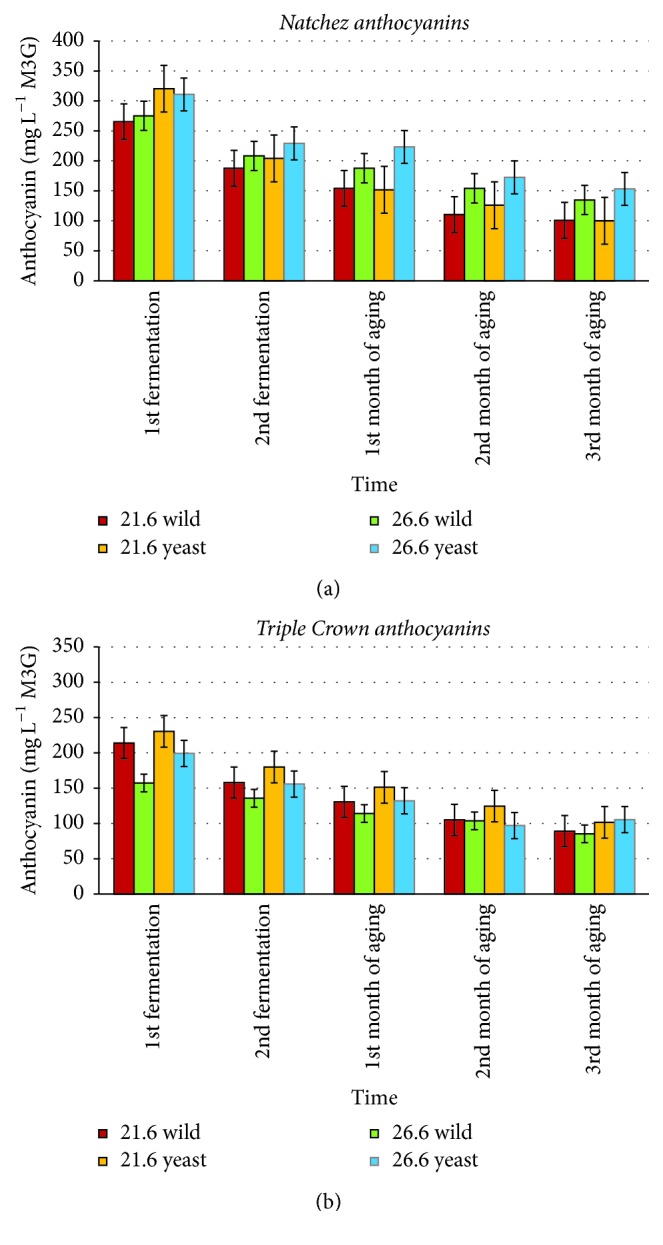
Total anthocyanin concentrations (*N* = 120) of “Natchez” blackberry (a) and “Triple Crown” blackberry (b) fermented juices and wines. Error bars represent ± standard deviation of the mean (*n* = 2).

*(2) Anthocyanin Concentration*. Anthocyanin concentrations of blackberry fermented juices and wines are shown in Figure 3. Anthocyanin concentrations are expressed as mg L^−1^ malvidin 3-monoglucoside (M3M). The anthocyanin concentrations of this study ranged from 85 to 320 mg L^−1^ M3M.

Our results showed somewhat higher anthocyanin concentration than some other reports (Figure 3). A research [34] showed that the range of anthocyanins was from 12 to 167 mg L^−1^ M3M in blackberry wine, which was twofold lower than our result. It is important to note that these wines were prepared using different methods compared to the wines processed in the current study. It is possible that the fermentation method used in the current study affected anthocyanin concentrations of blackberry juice and wine. The blackberry cultivars used were different as well.

Statistically significant differences were found between cultivars, fermentation temperatures, and inoculation levels (*p* < 0.05). In this study, wines made from “Natchez” berries showed higher anthocyanin concentrations in the berries than wines made from “Triple Crown” berries. “Natchez” berries were relatively large and soft-skinned. They may have broken down more easily at the higher fermentation temperature and this may have led to a more complete extraction of anthocyanins. On the other hand, “Triple Crown” berries were relatively small and firm. They may not have broken down as well and thus the longer fermentation time seen at the lower fermentation temperature may have allowed a more complete extraction. For inoculation levels, yeast inoculation samples had higher anthocyanin concentration than wild fermentation. Yeast-inoculated samples may helped to break down polymeric pigments and provide higher anthocyanin concentration.

Overall, the anthocyanin concentration decreased during wine aging, likely due to polymerization and copigmentation. During storage, anthocyanins gradually disappear as monomeric compounds and are transformed into polymeric forms, resulting in loss of color [35]. Also, newly formed pigments derived from chemical reactions between anthocyanins and various wine compounds, including phenolic compounds, could contribute to the color characteristics of aged wines. It has been suggested that higher temperature extraction could accelerate formation of new, more stable wine pigments [3, 24, 36].

#### 3.2.2. Oxygen Radical Absorbance Capacity (ORAC)

**Figure 4:**
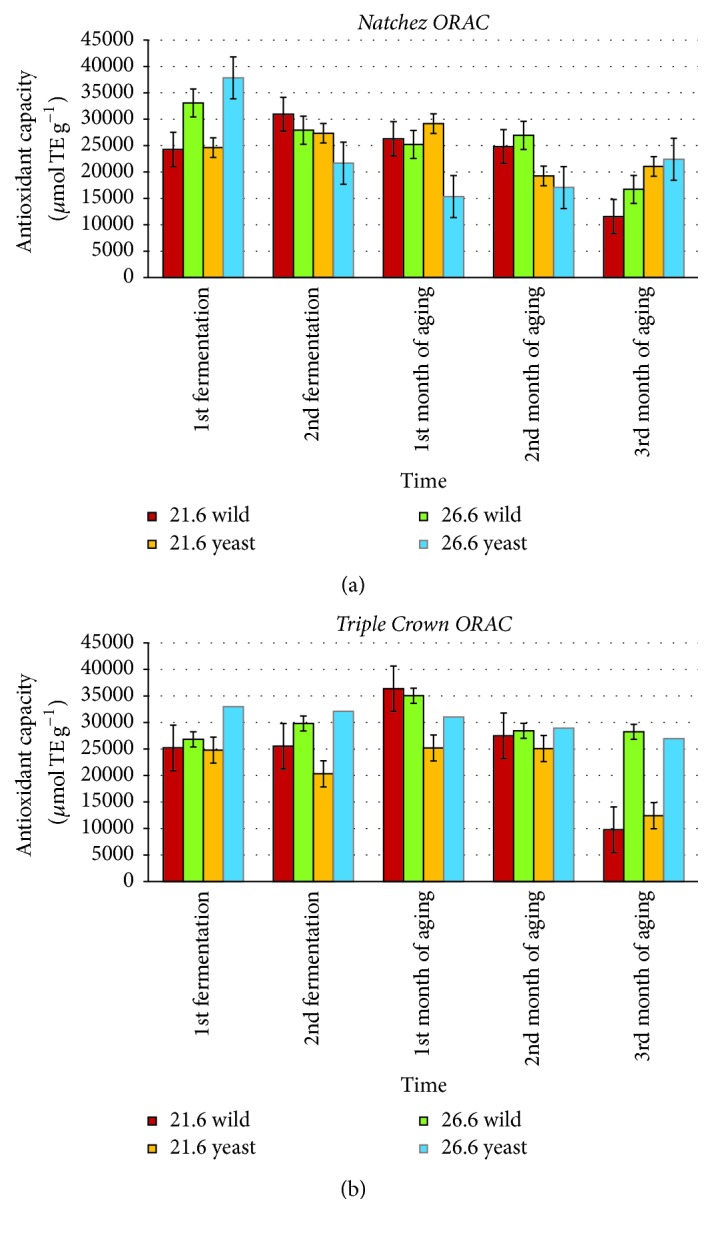
Oxygen radical absorbance capacity (*N* = 120) of “Natchez” blackberry (a) “Triple Crown” blackberry (b) fermented juices and wines. Error bars represent ± standard deviation of the mean (*n* = 2).

The ORAC values for blackberry fermented juice and wines are shown in Figure 4. The range of ORAC values observed in this study was between 9776 and 37845 *µ*mol Trolox equivalent (TE) g^−1^. Overall, cultivar and fermentation temperature had the greatest influence ORAC values, but the effects were not consistent over time.

The average mean ORAC values of blackberry pomace are shown in Table 3. Values ranged from 16625 to 23200 TE g^−1^. “Triple Crown” pomace generally showed higher ORAC values than “Natchez” pomace. For pomace samples, blackberry seeds and skin residue had higher antioxidant activity than aged wine samples, indicating that the pomace retains substantial amounts of antioxidant compounds even after wine processing. In general, wild fermentation samples showed higher antioxidant capacity than yeast-inoculated samples. Samples with higher fermentation temperature showed statistically significant differences (*p* < 0.05).

As with the anthocyanin concentrations previously reported, the ORAC values recorded in this study (Figure 4) were somewhat higher than those reported in other literature. A Spanish researcher [2] showed that the range of ORAC values observed in blackberry juices was 39160 *µ*mol TE g^−1^. This range was close to our result but slightly higher. Other articles [32, 37] showed that the ORAC values varied a great deal depending on variety, geographic growing location, and extraction method. As fermentation techniques and extraction methods are known to influence ORAC values, these results may indicate that the Korean fermentation style used in this study was more efficient in extracting and/or preserving antioxidant activity in the final wine than some other fermentation methods examined in previous studies.

Fermentation temperature influenced the antioxidant capacity in blackberry juice and wine in this study (Figure 4), but the results were not consistent over time. At times, “Natchez” wines showed higher antioxidant activity at the lower fermentation temperature and “Triple Crown” wines showed higher antioxidant activities associated with the higher fermentation temperature. At other times, the reverse was true. By the end of the three-month aging process, higher antioxidant activity was generally correlated with higher fermentation temperatures for both cultivars and inoculation treatments. Similarly, the influences of cultivar and inoculation type on ORAC values (Figure 4) were not consistent over time. By the end of the three-month aging process, no clear pattern for the effects of cultivar and/or inoculation type on antioxidant activity was discernable.

Overall, there was not clear pattern of correlation between ORAC, total phenolic concentration, and anthocyanin concentrations. However, common patterns in ORAC values and total phenolic concentrations were seen with respect to cultivar and fermentation temperature. One possible cause for the mixed results seen overall for correlations among ORAC values, anthocyanins, and total phenolic concentrations could be variations in cultivar genetics. As noted previously, the two cultivars had notably different berry tissue consistencies and this could have influenced the relative efficiency of phenolic extraction overall and among fermentation temperature treatments.

Also, it is important to note that other compounds may have had a significant influence on the measured ORAC values. Anthocyanins are one class of phenolic compounds, but other types may have had a greater or lesser impact on measured ORAC values over time as the aging process progressed and various polymers and complexes evolved. Further research would be required to elucidate the relationship between all of the compounds present in the wine and the observed antioxidant capacity. In addition, the ORAC test employed in this study provides a relative quantification of the wines' ability to quench peroxyl radicals; other antioxidant tests could be employed in future research to measure antioxidant capacity relative to other oxygen radical species. This could demonstrate additional correlations.

## 4. Conclusion

This research showed that the blackberry wines made in this study were relatively high in polyphenolic compounds as well as antioxidant capacity. As the Korean wine making method is of relatively low cost and easy to adapt to small-scale production, it may be especially well suited to helping small local growers and/or processors to add value to the blackberry crop such as “Natchez” in the Midwestern United States.

## Figures and Tables

**Table 1:** Average mean concentration of pH, titratable acidity, and soluble solid values of pressed blackberry juice (*n* = 2).

Cultivars	Natchez		Triple Crown	
Harvest year	2011	2012	2011	2012
pH	2.88	3.01	2.92 a^a^	3.15 b
Titratable acidity (% malic acid)	0.386	0.403	0.391 a	0.422 b
% soluble solids	11.64 a	10.04 b c^b^	11.08	12.06 d

^a^Means with a and b letters indicate significant differences between years (*p* < 0.05). ^b^Mean with c and d letters indicate significant differences between cultivars (*p* < 0.05).

**Table 2:** Mean values of pH, titratable acidity, and % alcohol in blackberry wines by inoculation type and fermentation temperature (*n* = 3).

Cultivar	Natchez				Triple Crown			
Inoculation treatment	Yeast		Wild		Yeast		Wild	
Fermentation temperature (°C)	21.6	26.6	21.6	26.6	21.6	26.6	21.6	26.6
pH	3.1 a^a^	2.6 b c^b^	3.08 a	2.64 b c	3.12 a e	2.87 b d e	3.05 a f	2.76 b d f
Titratable acidity (% malic acid)	0.42 a	0.35 b c	0.41 a	0.35 b c	0.42 a e	0.39 b d e	0.41 a f	0.37 b d f
% alcohol	13.91 c e^c^	13.57 c	13.46 f	13.26 c	15.76 a d e	15.01 b d e	13.68 f	13.67 d f

^a^Means with a and b letters indicate significant differences between fermentation temperatures within cultivar and inoculation treatment (*p* < 0.05). ^b^Means with c and d letters indicate significant differences between cultivars within inoculation treatment and fermentation temperature (*p* < 0.05). ^c^Means with e and f letters indicate significant differences between inoculation treatments within cultivar and fermentation temperature (*p* < 0.05).

**Table 3:** Mean ORAC values for blackberry pomace (*n* = 3).

Cultivars	Natchez				Triple Crown			
Inoculation treatment	Yeast		Wild		Yeast		Wild	
Fermentation temp. (°C)	21.6	26.6	21.6	26.6	21.6	26.6	21.6	26.6
ORAC (*µ*mol TE/g)	16920	18628 a^a^	16625	19055 a	17209 c^b^	12481 b d e^c^	19428	23200 b f

*N* = 24. ^a^Means with a and b letters indicate significant differences between cultivars within fermentation temperature and inoculation treatment (*p* < 0.05). ^b^Means with c and d letters indicate significant differences between fermentation temperatures within cultivar and inoculation treatment (*p* < 0.05). ^c^Means with e and f letters indicate significant differences between inoculation treatments within cultivar and fermentation temperature (*p* < 0.05).
